# Awareness and Information Seeking About Bowel Cancer and Screening in a Remote Australian Community: A Qualitative Study

**DOI:** 10.1111/hex.70769

**Published:** 2026-07-15

**Authors:** Nicola Gadd, Simone Lee, Jessica Hughes, Matthew J. Sharman, Ha Hoang, Kehinde Obamiro

**Affiliations:** ^1^ Centre for Rural Health, School of Health Sciences University of Tasmania Launceston Tasmania Australia; ^2^ School of Medicine University of Tasmania Hobart Tasmania Australia; ^3^ School of Psychological Sciences University of Tasmania Launceston Tasmania Australia; ^4^ School of Health Sciences University of Tasmania Launceston Tasmania Australia; ^5^ Central Queensland Centre for Rural and Remote Health James Cook University Emerald Queensland Australia

**Keywords:** awareness, bowel cancer, early detection of cancer, Information seeking behaviour

## Abstract

**Introduction:**

Understanding how individuals find bowel cancer and screening information and what influences awareness may support targeted interventions and encourage informed participation in screening programmes and support early detection. This study aimed to identify factors that influence remote community members information‐seeking behaviours and awareness of bowel cancer and screening.

**Methods:**

A qualitative study was conducted with 16 community members aged 50‐years and over living in a remote Tasmanian community in Australia. Semi‐structured interviews were informed by the Theoretical Domains Framework and Behaviour Change Wheel. Interview questions were reviewed by three consumers with lived experience of bowel cancer and screening to improve clarity and accessibility. Interviews were analysed using qualitative content analysis with inductive and deductive coding.

**Results:**

Information‑seeking was shaped by self‑efficacy, health literacy, personal relevance, and social influences, with trusted healthcare providers playing a central role. Awareness influenced screening decisions, while screening experiences also built awareness across the screening pathway to diagnosis. Some participants screened with an understanding of the risks and benefits, while others complied without understanding why, and some with low awareness chose not to screen. Awareness most often developed through passive information exposure, including environmental cues, screening material prompts, and social influences of routine healthcare encounters, and conversations with family, friends, and healthcare providers, rather than through active information‑seeking.

**Conclusion:**

In this remote community, bowel cancer awareness was commonly shaped through passive exposure rather than deliberate searching and influenced the quality of screening decision‑making. Health promotion strategies that embed accessible, plain‑language information within everyday community and healthcare settings, and leverage trusted relationships, may better support informed and equitable participation in bowel cancer screening than approaches that rely on active information‑seeking.

**Patient or Public Contribution:**

Consumers with a lived experience of bowel cancer and screening contributed to this study by reviewing the interview guide structure and questions prior to the researchers undertaking the interviews. This improved the plain language and the participants understanding of the questions, with the addition of an introductory script outlining the study aims and process of the interview. The final interview guide was sent to the consumers for approval prior to the first interview being conducted. These consumers did not participate in this study. Remote Tasmanian community members who were eligible or previously eligible to participate in the National Bowel Cancer Screening Program contributed to this study as participants who shared their experiences and insights into bowel cancer and screening information‐seeking behaviours and awareness.

**Abbreviations**
BCW: Behaviour Change Wheel
GP: General Practitioner
NBCSP: National Bowel Cancer Screening Program
SPSS: Statistical Package for the Social Sciences
SRQR: Standards for Reporting Qualitative Research
TDF(v2): Theoretical Domains Framework, version 2

## 1 Introduction

The highest bowel cancer mortality rates in Australia, are in Tasmania (30.4 per 100,000, 2019–2023), a state comprising of inner regional to very remote areas, with no major cities [1, 2]. In Australia, the National Bowel Cancer Screening Program (NBCSP) is available for those aged 45–74 years and involves completing a screening kit sent in the mail every 2 years [3]. The programme aims to encourage early detection and reduce mortality rates, although across Australia, screening rates are low (42.0%, 2023–2024), especially in rural and remote areas (41.7% major cities; 26.5% very remote) [1]. Tasmania has the highest screening rates in the country (46.7% 2023–2024, alongside South Australia), but participation has declined (48.2%, 2019–2020) [1]. Along with the decline in screening and high mortality rates, the median wait time between a positive screening test and a diagnostic assessment in Tasmania (82 days) is above the national average (62 days) [1]. This is consistent with other rural (69 days) and remote areas (80 days) in Australia having higher wait times compared to major cities (58‐days), which may delay diagnosis and outcomes for these areas [1]. Strategies to improve screening rates are needed, particularly in rural and remote communities where inequities in cancer outcomes persist [1]. Tasmania presents as an exemplar for such rural and remote bowel cancer research focus in Australia, with little research in this area prior to this project.

Awareness and information‐seeking are important determinants of screening engagement, but they do not operate in isolation. In rural and remote communities, health‐seeking is shaped by broader structural and contextual factors, including geographic distance, limited service availability, workforce shortages, transport difficulties, and poorer access to primary healthcare [4, 5]. These broader rural access barriers are also evident across the cancer pathway [1]. People living in rural and remote areas may experience reduced access to screening and cancer care, travel burdens associated with specialist services, and delays in diagnosis or follow‐up assessment after a positive screening result [1]. Social and attitudinal factors may further influence screening engagement [6]. Goodwin et al. [6] reported that environmental and cultural factors in non‐metropolitan areas may support more reactive approaches to healthcare, and that attitudinal and cognitive traits including fatalism, self‐reliance and consideration of future consequences, were associated with bowel cancer screening compliance [6]. Therefore, this study considers awareness and information‐seeking as inter‐related with broader rural access, social and behavioural influences, rather than as isolated determinants of screening behaviour.

Awareness of bowel cancer symptoms, risk factors and screening options is important for encouraging eligible individuals to participate in preventative health behaviours, including screening [7]. For example, understanding the risk of higher levels of daily alcohol (> 3 standard drinks) increases one's chance of bowel cancer, may encourage individuals to reduce their intake [7, 8]. This is particularly relevant in Tasmania, where in 2022, 9% of adults reported drinking daily and 36% reported weekly consumption [9]. However, evidence suggests that awareness of bowel cancer among Tasmanians is limited. Lee et al. [7] identified that many Tasmanians had poor awareness of risk factors and symptoms, with over half unable to confidently identify a symptom [7]. Similarly, Gadd et al. [10] reported low levels of perceived bowel cancer information exposure among rural and remote Tasmanians, suggesting limited bowel cancer information may be available in these areas. Such information about risk factors and symptoms should be known so individuals can make informed decisions about their health.

Awareness of bowel cancer screening may also improve individuals' awareness of bowel cancer risk factors or symptoms [7]. Jalleh et al. [11] found participants who were aware of NBCSP were more likely to correctly identify key symptoms, such as blood in stools or changes in bowel habits [11]. This suggests that educating individuals on risk factors, symptoms and screening options may encourage early detection through screening and improve bowel cancer related morbidity and/or mortality.

To raise awareness, it is important to understand how individuals find bowel cancer information. Existing literature mostly focused on cancer patients/survivors’ information‐seeking as coping mechanisms following diagnosis, rather than public seeking information to increase awareness and screening [12, 13]. There is also limited literature on bowel cancer specific information‐seeking behaviour in Australian settings [14, 15, 16]. A US study suggested participants commonly obtained bowel cancer information both actively and passively through interactions with doctors, television/radio and internet [17]. Another US study reported that among individuals who searched for cancer information, some experienced frustration, questioned the quality of information and/or felt the process took much effort [14]. Those with negative information‐seeking experiences were also more likely to report beliefs such as ‘not much can be done to prevent cancer’ or ‘it is hard to know which cancer prevention recommendations to follow’ [14]. This suggests that confusion may arise from the information‐seeking process and some individuals may require support in finding appropriate cancer‐related information [14].

There is limited research in Tasmania, and Australia more broadly, examining how people in rural and remote communities are aware of bowel cancer and their information‐seeking behaviours. Understanding these processes is critical for designing community‐appropriate strategies that support informed decision‐making and equitable access to preventive health information. This study aimed to explore factors influencing remote Tasmanian community members’ experiences of seeking bowel cancer and screening–related information and their awareness.

## 2 Methods

This qualitative study interviewed remote Tasmanians to identify factors influencing bowel cancer screening participation (NBCSP), information‐seeking behaviours and awareness. The current paper reports on the analysis of factors influencing bowel cancer information‐seeking behaviours and awareness of bowel cancer and screening. The analysis of screening participation factors was reported in Gadd et al. [18]. This paper was reported in accordance with Standards for Reporting Qualitative Research (SRQR) checklist [19].

### 2.1 Design

This qualitative study was informed by the Theoretical Domains Framework for behaviour change version 2 (TDF(v2)), Behaviour Change Wheel (BCW), and Integrated Screening Action Model (I‐SAM) [20, 21, 22]. The TDF(v2) was selected to support systematic identification of barriers and enablers affecting behaviour with consideration of relevant domains which may influence bowel cancer information‐seeking and screening‐related behaviour [20]. The BCW, including the COM‐B model, was used to understand behaviour as arising from interactions between capability, opportunity and motivation [21]. As information‐seeking and screening are behaviours, the TDF(v2) and BCW were used to guide the development of the interview guide (Appendix 1) and to support analysis of information‐seeking data [20, 21]. Awareness was conceptualised as a factor that may influence screening‐related behaviour, rather than as a behaviour itself. Therefore, I‐SAM [22] was additionally used to support the organisation and interpretation of awareness findings. I‐SAM incorporates the BCW [21] and conceptualises screening engagement as a process shaped by individual and environmental influences, helping to identify potential intervention opportunities across the screening pathway [22]. Semi‐structured interviews with open‐ended questions were used to explore participants’ experiences with screening, their awareness of bowel cancer and their approaches to seeking related information. This allowed the researchers to explore barriers and enablers for bowel cancer awareness and information‐seeking. To ensure the questions could be easily understood by participants, three consumers with lived experiences of bowel cancer and screening reviewed the draft of the interview guide before data collection. They provided feedback on the clarity, plain‐language wording and structure of questions. The interview guide was revised in response to this feedback, including simplifying wording, adding an introductory script outlining the study aims and interview process, and adding further prompts to support participant understanding. The final interview guide was then provided to consumers for review before the first interview. These consumers did not participate as interview participants in the study.

### 2.2 Participants and Recruitment

Participants were adults aged 50‐years‐old and over (including those who were older than the NBCSP routine invitation age range) from one Tasmanian remote community. The interview guide was not modified according to participant age. However, questions were phrased broadly to capture diverse screening experiences, including participation, non‐participation, prior screening, colonoscopy, and experiences of no longer receiving NBCSP kits. The community was chosen for a strength‐based approach, as it had the highest bowel cancer screening rates in Tasmania in 2018‐19 (54.8%) [23]. A strength‐based approach allowed the researchers to understand the strengths of that community which encouraged screening, to inform health promotion strategies for other areas with lower screening rates [24]. To ensure participants understood the study requirements, individuals with cognitive impairment were excluded. Cognitive impairment was tested with the ‘Single‐question test on progressive forgetfulness (patient)’ [25]. Participants were recruited from July to December 2022 until data saturation, where no new information emerged from the last three interviews. Interviews ran between September to December 2022. Participants were recruited through purposive sampling and snowballing, where participants referred eligible community members to participate. Flyers were advertised in the community and the communities’ general practitioners and pharmacists referred eligible individuals to participate. Healthcare providers ensured eligibility through checking the patient's age and medical history (screening status, cognition) through their records. To minimise the feeling of coercion, healthcare providers were asked to provide eligible individuals with the flyer or information sheet and encourage individuals to contact researchers directly, without disclosing their interest with the provider. Individuals who were recruited through participant referrals and advertisements all completed the cognitive impairment test to check eligibility.

### 2.3 Data Collection and Analysis

A paper‐based participant demographic questionnaire was completed at the start of each interview, including questions about age, sex, education and history of bowel cancer and screening (see Appendix 2 for questions). Semi‐structured interviews were conducted face‐to‐face or via videoconferencing. Interviews lasted 30‐60 min, were audio‐recorded and transcribed verbatim, with written field notes.

Data was analysed by two researchers using both directed (deductive) and conventional (inductive) content analysis [26]. Data was analysed to identify participants’ views and experiences of barriers and enablers to seeking bowel cancer and screening information and awareness. Transcripts were cleaned to ensure consistent formatting and de‐identified before analysis. Two researchers independently read the preliminary data once without coding, then reread the transcripts to code sections relevant to awareness and information‐seeking. Coding included inductive coding to capture concepts emerging from the data and deductive coding informed by relevant TDF(v2) domains [20, 27].

Codes were subsequently mapped as barriers or enablers of information‐seeking behaviours and awareness. For information‐seeking, coded data were grouped deductively into themes based on the BCW, where TDF(v2) domain codes directly mapped to COM‐B components of capability, opportunity and motivation [21], as outlined in Atkins et al. [20]. In contrast, awareness related codes were developed inductively and then organised using I‐SAM [22] informed analytical lens, supported by the BCW [21], to reflect how awareness developed and influenced screening‐related decisions across the screening pathway. Awareness was conceptualised as a domain that could ‘influence’ behaviour rather than a behaviour itself, consistent with TDF(v2) ‘knowledge’ domain [20]. Thus, while both awareness and information‐seeking data was coded using inductive and deductive approaches, the development and organisation of themes differed. NVivo (V.12, 2020) [28] was used to store transcripts and manage coding during analysis. Demographic data was analysed descriptively using SPSS [29].

### 2.4 Techniques to Enhance Trustworthiness

Two researchers independently coded the data, and discussed preliminary codes for consistency, to ensure agreement of the code's meanings. A coding system adapted from TDF(v2) domains [20] was developed to minimise risk of bias (Appendix 3). Disagreements of the code's meanings were resolved via consensus or from input of a third researcher. Participants were offered the opportunity to review their transcripts following transcription to correct errors or to clarify sections [30]. Transcripts were provided via email to all six participants who agreed to this opportunity, with no corrections or clarifications requested from participants.

### 2.5 Ethical Approval

Participant consent was obtained, and ethical approval was sought from University of Tasmania Human Research Ethics Committee (approval number: H0027256).

## 3 Results

Sixteen participants completed interviews. Of those, 81.0% were female, 31.0% had a medical history of bowel cancer and/or polyps and 6.3% had never completed a stool test (Table 1). Themes were reported separately for bowel cancer and screening information‐seeking behaviours and awareness. The three themes for information‐seeking were based on BCW components which can influence a behaviour: capability, opportunity and motivation [21]. Bowel cancer and screening awareness included three themes: 1. Social influences enabled passive exposure to bowel cancer information and raised awareness of bowel cancer and screening. 2. Environmental cues and screening materials prompted awareness, but understanding of bowel cancer and screening was variable and incomplete. 3. Awareness influenced screening decisions, while screening experiences also built awareness across the screening pathway. See Appendix 4 and Appendix 5 for summary tables of the themes and sub‐themes.

**Table 1:** Participant demographics and characteristics [(*n* = 16); *n* (%)].

Age (years)	
50–54	4 (25)
55–59	2 (12.5)
60–64	2 (12.5)
65–69	3 (19.0)
70–74	4 (25.0)
75+	1 (6.0)
Sex	
Male	3 (19.0)
Female	13 (81.0)
Other	0
Highest level of education	
Primary school	0
High school	1 (6.3)
Year 12	1 (6.3)
TAFE/trade	9 (56.0)
University	5 (31.0)
Employment status	
Employed	10 (62.5)
Unemployed	0
Retired	6 (37.5)
Previously had colonoscopy	
Yes	7 (44.0)
No	9 (56.0)
Number of stool tests completed previously	
0	1 (6.3)
1	4 (25.0)
2	4 (25.0)
3	1 (6.3)
4+	6 (37.5)
History of bowel cancer	
Yes	5 (31.0)
No	11 (69.0)
Family history of bowel cancer	
Yes	11 (69.0)
No	5 (31.0)
Number of people live with	
1	3 (19.0)
2	9 (56.0)
3	3 (19.0)
4+	1 (6.3)

### 3.1 Bowel Cancer and Screening Information‐Seeking Behaviours

#### 3.1.1 Theme One: Capability of Bowel Cancer Information‐Seeking Behaviour

This theme involved how individuals’ physical skills and psychological capability can influence bowel cancer information‐seeking [21].

##### 3.1.1.1 Physical Skills

Self‐efficacy was identified as both a barrier (low self‐efficacy) and an enabler (high self‐efficacy) to look for bowel cancer information. ‘I've just asked Google about information on bowel cancer’ (#8). Enablers for information‐seeking were finding it easy to look or believing that they knew how to find the information. ‘Have no issue, looking for any information. If you're computer literate, that's an advantage’ (#9). Barriers to seek information were having low confidence to search for the information (poor internet and literacy skills). ‘Me and my skills. Probably not… I would have to go to the library and do it the hard way [ask librarian for support]’ (#3).

##### 3.1.1.2 Psychological Capability

Bowel cancer being personally relevant to someone could enable them to pay more attention to such information e.g., family history, knowing someone with bowel cancer or for their own health, ‘if you're feeling well… why would you look?’ (#1). ‘Talking to people with experience that have gone through it, or we've had family members go through it, makes you think about it, and then find out a bit more’ (#1).

Some discussed that people may not take notice of bowel cancer information if they have low awareness or if there are no triggers to capture attention. ‘Watch things on TV… doesn't mean it's… up to the individual… whether you do it or listen’ (#11). Dyslexia, a learning disorder was identified by one participant as a barrier for sourcing information.

Have got dyslexia, so it's quite wordy [screening kit instructions]. Maybe it needs… simpler… for someone that has… literacy issues… pictorial with less word and in big print…. plain English, not too medical… if there's a whole heap of words on the page and you're struggling with your literacy… you're just not gonna bother.

(#10)

#### 3.1.2 Theme Two: Opportunity of Bowel Cancer Information‐Seeking Behaviour

This theme discussed factors external from the individual (social and physical environment) that prompt or make information‐seeking possible [21].

##### 3.1.2.1 Social Factors

Social influences only enabled people to seek information. ‘Bloke's group, we get together a couple of times a week… talk about things…. prompts you to think… maybe I should, find out a bit more’ (#1). ‘Friends, their mothers have died. And fathers have died through bowel cancer. That makes you a little bit more inquisitive… more aware and to look up, to make sure you don't have the symptoms’ (#2). Family, friends, or healthcare providers can either pressure others to look or can be the source of information. ‘Family history and my sister getting on my case about it’ (#5). One participant shared how they looked for information to motivate their partner to screen. ‘Husband getting his [NBCSP] kit first and him refusing… definitely encouraged me to go looking for more information and, certainly trying to show him more information… to say… this isn't so bad’ (#15).

##### 3.1.2.2 Physical Environment

Participants discussed finding information both intentionally (actively seeking) and unintentionally (passive seeking). Participants mostly reported finding information unintentionally, by stumbling across information through printed materials or television/radio. ‘Haven't deliberately sought out information about it [bowel cancer]. But I do stuff like, read the wellness pieces in the newspaper’ (#12). Ways people intentionally sought information were through the internet, speaking with healthcare providers or by receiving the NBCSP kit which triggered them to look for more information. ‘Google it, comes up with all the sites. But knowing what site to look at is really important’ (#9). The reliability of the information participants found on the internet varied, with some searching reputable websites whereas others accessed information from the first page of search results.

#### 3.1.3 Theme Three: Motivation of Bowel Cancer Information‐Seeking Behaviour

This theme discussed the automatic (impulsive, emotional) or reflective processes (planning and evaluating) that motivate individuals to seek (or not seek) bowel cancer information [21].

##### 3.1.3.1 Automatic Processes

Having an interest in reading about health or to help others were enablers for people to find/seek information. ‘Haven't, sought out any information specifically. My knowledge of the risk factors just come through… general reading’ (#7). Participants who worked in healthcare looked for information to help others understand that bowel cancer is treatable and to reduce their fears. ‘I have sought information about bowel cancer recommendations, especially when counselling others [healthcare provider] so, they've got facts’ (#9).

Some may not look for bowel cancer information due to denial or not wanting to know, ‘I'd rather not know…. That's what [partner] says. Me. I'd rather know’ (#13). ‘I'm not a seeker… [do not look for information]’ (#3). Some discussed that ‘older people tend to be much more private. That was the way it always was in their generation… didn't discuss [health] with anyone, you probably didn't even go and see your doctor about it, because they were things that you just kept very private’ (#5). They suggested that if there were more positive attitudes towards bowel cancer (less taboo), individuals may be more inclined to seek information.

##### 3.1.3.2 Reflective Processes

Having personal relevance (symptoms, personal or family history) motivated some to look for information. ‘Whole family basically, looking into… [bowel cancer] that's when I approached my GP to get my first colonoscopy organised, because… risk of the [bowel cancer] family history’ (#5). Some sought information to make better health choices and have control over their potential health outcomes.

The days have gone where we need to rely on the medical profession to tell us what's happening about our health… we need to know more about our own bodies, our own health, so that we can be a bit more proactive [to make changes].

(#5)

A lack of personal relevance or interest to look were barriers, as some reported it not necessary to look or did not look because they regularly screened and lacked symptoms. ‘I just do the screening at the moment [not looking for information], unless I notice some of those symptoms… then of course I would [look]’ (#10). Others believed healthcare professionals took care of it and told them what they needed. ‘I've got enough understanding… I'll leave the technical stuff to the specialists’ (#6). Some intended to live a healthy life for overall health, and reduce their risk of all cancers, rather than worrying about one type.

I've been more concerned about trying to live a healthy life, and not really hone in on one sort of ailment. To me, [to prevent] cancer in general, we just try and keep as healthy as you can, and eat properly and exercise and don't smoke, don't drink, all those sorts of things, and hope that they'll all stay at bay.

(#15)

Some participants would draw on family experience for awareness instead of seeking information. ‘Haven't… sought any information, I just go by what I've seen with family’ (#13). Some thought people cannot be forced to seek information; they needed to want to do it or see value. ‘Have to want to seek it out. You can't be made to seek it out’ (#16).

### 3.2 Bowel Cancer and Screening Awareness Influences

The findings suggested that awareness was shaped by social influences, environmental cues, screening materials and screening‐related experiences. These factors affected whether participants developed sufficient understanding to recognise the personal relevance of bowel cancer screening and make informed screening decisions.

#### 3.2.1 Theme One: Social Influences Enabled Passive Exposure to Bowel Cancer Information and Raised Awareness of Bowel Cancer and Screening

Social influences in this theme aligned with BCW opportunity social [21] and I‐SAM model [22] social influencers on screening behaviour including social support, health provider endorsement and mass media. Social support enabled awareness for remote Tasmanians, through knowing someone with bowel cancer and regularly having conversations with others. One participant discussed how they were aware the screening kit process was easy after discussing it with their partner, ‘from my partner, I understand it's very easy’ (#14). Knowing someone with lived experience of bowel cancer (friend or family) can increase one's awareness of symptoms, the cancer journey or risk factors – through other peoples’ experiences and/or discussions with them. One participant shared their awareness of the bowel cancer journey through a friend's experience. ‘A friend… was diagnosed with colon cancer and had to have a section of the colon removed… Which certainly has surprised me, I didn't realise it at the time’ (#7). Some participants assumed what the symptoms were based on their family's or friends’ experiences. Although, participants with family history often had good knowledge of bowel cancer. ‘My aunt had bowel cancer… polyp's blockages in the bowel, having to have a resection of your bowel and a bag’ (#2).

General practitioners were reported as important in raising awareness, but their lack of time to do so in consults was a barrier. ‘Doctors are a really important part of this… [discussing screening] problem is that there isn't time to introduce much bigger… picture stuff in normal interactions with their patients’ (#14). When asked about screening, some referred to an existing awareness campaign, ‘something we need to be more aware of… poo, poke, and post’ (#13). Awareness strategies were reported as needed for younger people to be made aware of screening, symptoms and risk factors, to start thinking about it. Opinions differed for what the age should be to focus on raising awareness. Generally, participants agreed it should start before people become eligible to screen, with some suggesting as early as adolescents or thirties. Participants believed that younger people should be aware of symptoms and risk factors to prevent complacency and for impactful behaviour change (reduce risk). ‘‘That's not gonna happen to me, I'm only 19’… more awareness to them’ (#13). Earlier awareness could prepare people for how and when to screen and to reduce the shock they may experience when they first become eligible and receive the NBCSP kit.

#### 3.2.2 Theme Two: Environmental Cues and Screening Materials Prompted Awareness, but Understanding of Bowel Cancer and Screening Was Variable and Incomplete

Environmental cues were consistent with BCW [21] opportunity physical and I‐SAM [22] physical environmental influences including prompts and invitation reminders. Being in the right environment (working in healthcare or in healthcare waiting room) can allow people to pay more attention to information. In a waiting room could spark one's curiosity to read from their surroundings or to find information unintentionally (rather than actively seeking). ‘Sister who had breast cancer. I probably have had time in hospitals waiting rooms and… being in the environment where I'm thinking of those kinds of things’ (#12). Whereas lack of information prompts to trigger attention were identified as barriers to awareness. Participants suggested prompts were needed to remind those who would not actively seek information about bowel cancer, as there was not enough information available. As having information visible could prompt people to think about bowel cancer. ‘[NBCSP] need to get onto some sporting identity or something… to get it out there. To make it… an everyday thing that you hear about, read about, see’ (#3). One participant reported that because bowel cancer was a taboo subject, often people would not think about it.

The screening kit was a physical prompt for some to think about bowel cancer. ‘It took actually getting the kit in the mail to make me even stop and think about it… certainly not something that I would have ever thought about… unless that kit came and prompted’ (#15). Some participants became aware of symptoms through reading information from screening kits or colonoscopies. ‘Bleeding is I think a prime one, because that's what this test [screening kit] looks for’ (#7). Although for some, receiving the kit was not enough to prompt as they did not open the kit, ‘send out a pack. Which has been left unopened… then lost’ (#16).

Participants discussed concerns of lack of information prompts from NBCSP other than the kits or after they are no longer eligible. Those older than NBCSP screening eligibility discussed concerns of no longer receiving kits and the lack of information regarding where to source alternative options. ‘Neighbours… they're 75, and they're a bit worried how they think they should still be entitled to [NBCSP] if they want it, but I believe they're not after 75… I don't know how you get over that’ (#4). Some older participants were proactive and actively sought this information from their doctor, ‘I did that [screening kit] until… 74 and then that's it. I haven't had it back. I raised this issue with my GP… he gave me a kit’ (#7). Whereas others felt older people may not openly talk about it and would not be proactive and ask if information was not directly provided to them. Similarly, one participant expressed concern that they had un‐registered from NBCSP when they first received the kit, but now they are older and more knowledgeable of bowel cancer, they wanted to re‐join. ‘I had for some years; been intending to get back to the bowel cancer screening group to say… I will become included [re‐register]’ (#14). They found the process difficult to re‐register and suggested information prompting them could assist this process. Other participants similarly suggested they would benefit from reminders as they had forgotten to screen after receiving the kit.

‘Reminder might be useful… if I receive information or an email or letter, I normally respond to it, whereas I've let time go by thinking, I must do that. Then… life is busy, and things dropped to the bottom of the list of priorities’.

(#14)

Participants’ awareness of bowel cancer symptoms, risk factors and screening was variable and often incomplete, with gaps particularly around asymptomatic screening, options outside the NBCSP, re‐registering after opting out, and screening after colonoscopy.

#### 3.2.3 Theme Three: Awareness Influenced Screening Decisions, While Screening Experiences Also Built Awareness Across the Screening Pathway

This theme aligned with I‐SAM [22] screening process stage of decision making. A bi‐directional influence of awareness and screening was present, where completing screening can increase awareness and having awareness can encourage screening. Different levels of awareness of bowel cancer can influence screening behaviour. For example, having awareness of bowel cancer, screening or the outcomes/consequences of not screening enabled some participants to be more motivated to screen. ‘Because too many people die because they don't go and do it’ (#11). Other participants who had limited awareness of screening reported that they had screened, complying with health recommendations without understanding why. While other participants with low awareness may not have understood why they should screen and decided not to. This decision to not screen may have been due to not having symptoms or being unaware of risk factors relevant to them personally. For one participant, having low awareness of the importance to screen led them to un‐register from the screening programme.

I have been opting out of screenings. I feel that I'm a very healthy individual. I'm fit, I have a good diet, and the resources could be better spent on people who have a higher risk. So, I've opted out… I didn't feel I was at high risk, but I have more information now and see that perhaps it would be useful to participate.

(#14)

Awareness (or lack) of the screening process which involves completing the kit, followed by a diagnostic assessment could influence NBCSP screening kit participation. As participants who reported awareness that after undergoing a colonoscopy, they did not need to complete the NBCSP kit, reported they did not complete the next kit. ‘Probably 50% of the ones… sent [NBCSP kit], I have completed, the ones I haven't, [have] been because it's been, either just prior to or just after a colonoscopy’ (#6). Whereas those who were unaware of this process following a colonoscopy, reported they continued to complete the kit (unnecessarily) when it was next received.

Participants reported that awareness of bowel cancer was also important to take notice of symptoms when/if they arise, and to change behaviours that reduce their risk factors. ‘It's not always detected in its early stages. So, the more you know about it, the more informed you are, the better off you are’ (#1). Low awareness of what symptoms to look for was identified as a risk factor itself. ‘Lack of awareness is a risk… don't know, don't follow things’ (#4).

## 4 Discussion

This paper reported on factors that influenced how remote community members sought bowel cancer and screening information and what influenced their awareness. This study found that awareness influenced how participants made screening decisions, with information most often encountered passively rather than actively sought. Importantly, awareness most often developed through passive exposure to information, including environmental cues, routine healthcare encounters, and conversations with family and friends, rather than through deliberate searching.

Consistent with prior research, participants most often encountered cancer information through routine interactions and media rather than deliberately searching for it, and some described confusion or frustration when attempting to evaluate information quality [14, 17]. Studies using population datasets similarly showed that negative searching experiences are associated with uncertainty and fatalistic beliefs about cancer prevention, indicating the limits of strategies that depend on individuals to independently locate and appraise complex information [14, 17, 30]. Our findings are also consistent with Australian and international research linking health literacy and self‑efficacy to information engagement, where higher confidence and literacy facilitated active seeking, and lower literacy was associated with passive exposure and preference for interpersonal sources [15, 31, 32]. Our findings aligned with prior Tasmanian findings on limited exposure to bowel cancer information in rural and remote settings [10].

Extending this literature, our findings showed how passive exposure functions as the dominant pathway to awareness in a remote community, with environmental cues and social networks, particularly trusted healthcare providers serving as low‑effort, credible entry points to bowel cancer information [7, 10, 30, 31, 32, 33]. The current study added nuance by demonstrating that awareness influenced the quality of screening decisions, supporting informed participation for some, but also contributing to uninformed compliance or disengagement when awareness was limited [7, 11, 15, 30, 31, 32, 33].

Mapping findings to the BCW clarified how behavioural influences of capability, opportunity, and motivation interact in remote contexts [21]. Participants’ capability centred on self‑efficacy and literacy. Those who were comfortable with technology or information navigation reported fewer barriers, while others highlighted difficulties with dense text, complex screening kit instructions, and dyslexia, reinforcing the importance of accessible communication [15, 31, 32]. Opportunity to engage with information was primarily created through social networks and the physical environment such as men's groups, family reminders, and routine interactions with healthcare providers functioned as low‑effort on‑ramps to awareness. Motivation reflected both automatic responses (interest *vs.* avoidance) and reflective goals (proactivity *vs.* reliance on clinicians) [21]. Notably, several participants expressed trust in clinicians to guide them, illustrating how interpersonal relationships may offset limited individual information‑seeking capacity and highlighting the role of healthcare providers in shaping informed engagement [30, 31, 32, 33].

### 4.1 Implications for Policy, Practice and Research

These findings suggested that approaches relying primarily on individuals to actively seek and interpret bowel cancer information are unlikely to support informed and equitable participation in remote communities. Instead, bowel cancer awareness may be more effectively promoted by embedding accessible, plain language information within everyday community and healthcare environments, where passive exposure can prompt attention and discussion without requiring high individual effort.

Healthcare providers were consistently described as trusted sources of information, indicating an important opportunity for routine, opportunistic conversations about bowel cancer screening. Brief, non‐judgemental prompts from general practitioners, nurses, pharmacists, and practice staff, particularly during unrelated visits, may help translate passive exposure into personally relevant, credible guidance. In settings where continuity with a single clinician is limited [34], team‐based approaches and consistent messaging across services may help mitigate the effects of provider turnover and maintain patient trust. In line with BCW [21] and I‐SAM [22] intervention opportunities, screening, symptom or risk factor reminders for practice staff could be embedded within electronic record systems to prompt conversations e.g. when patients are close to receiving the screening programme kit or if have recorded symptoms or risk factors. With access limitations to health services in rural areas (travel, workforce shortages) [35], further strategies are needed to target those who have difficulty or do not engage with healthcare providers. Social connections and physical environmental cues may play such a role.

Social networks and community groups played a central role in normalising bowel cancer discussion and stimulating information engagement, particularly where bowel cancer remained a sensitive or taboo topic. Leveraging existing community spaces and peer networks, such as social groups, clubs, or informal gatherings may therefore support awareness by creating opportunities for conversation and shared understanding. Positioning bowel cancer information in these contexts as environmental prompts [21] for conversations may help shift responsibility away from individuals and towards community embedded support structures.

The findings also highlighted the importance of reducing cognitive and literacy related barriers to information engagement. Participants described difficulty navigating dense text, complex instructions, and medically framed language, suggesting a need for screening materials to prioritise plain language, visual cues, and step‐by‐step formats. Designing resources that accommodate varied literacy levels and learning needs may help ensure that screening participation reflects informed choice rather than compliance or disengagement.

Finally, participants’ concerns about screening eligibility, programme cessation, and re‐registration indicated the need for clearer communication across the screening life course. Proactive information at transition points, such as approaching eligibility, following colonoscopy, or exiting the national screening programme may help individuals understand available options and next steps, supporting continuity of informed decision making over time.

Future research should examine how bowel cancer awareness develops through passive information exposure across diverse rural and remote settings, particularly in communities with lower screening participation. Comparative studies across multiple locations may help determine the transferability of the mechanisms identified in this study, including the roles of environmental cues and social networks. Longitudinal or mixed‑methods studies could explore how awareness and information‑seeking evolve across the screening life course, including transitions into eligibility, programme exit, and re‑engagement.

### 4.2 Strengths and Limitations

This study has several strengths, including the use of a strength‐based approach and engaging with a single remote Tasmanian community. These allowed the study to explore in‐depth what worked well in that one remote community to encourage screening, the influencing factors and to identify information‐seeking behaviours in such community. The mechanisms identified, particularly passive information exposure, social influence, and trust in healthcare providers may be transferable to similar rural and remote contexts. The study was guided by established theoretical frameworks TDF(v2) [20], I‐SAM [22] and BCW [21], supporting systematic exploration of factors influencing awareness and information‑seeking. Consumer input into the interview guide structure enhanced accessibility and relevance, strengthening the credibility of findings. Methodological rigour/trustworthiness was supported through double‑coding, consensus discussions, and opportunities for participant transcript review [36].

There are also limitations to consider. Participants who volunteered may have had greater interest in bowel cancer screening than those who did not participate. Several had personal, family or close social experience of bowel cancer. These experiences were identified in the analysis as an influence on their information‐seeking and awareness and may have impacted the findings. Participants who had never screened were difficult to recruit and are under‑represented, potentially constraining insight into disengagement from screening.

## 5 Conclusions

Bowel cancer awareness in this remote community emerged predominantly through passive exposure and social interaction, shaping the quality of screening decisions. Fragmented awareness contributed to uninformed compliance or opting out while clearer understanding supported informed participation. Embedding plain‑language information within community and healthcare environments, supported by brief prompts from trusted healthcare providers, may reduce cognitive burden and improve equity in engagement. Designing passive‑to‑active pathways from environmental cues to timely, personalised guidance offers a practical approach to support informed participation across the screening life course.

## Author Contributions

**Nicola Gadd:** conceptualisation (supporting); data curation (lead); formal analysis (equal); investigation (equal); methodology (lead); project administration (lead); resources (lead); visualisation (lead); writing – original draft preparation (lead); writing – review and editing (lead). **Simone Lee:** conceptualisation (supporting); funding acquisition (equal); formal analysis (supporting); methodology (supporting); supervision (supporting); writing – original draft preparation (equal); writing – review and editing (equal). **Jessica Hughes:** data curation (supporting); formal analysis (equal); investigation (equal); writing – original draft preparation (equal); writing – review and editing (equal). **Matthew J. Sharman:** conceptualisation (supporting); formal analysis (supporting); methodology (supporting); supervision (supporting); writing – original draft preparation (equal); writing – review and editing (equal). **Ha Hoang:** methodology (supporting); supervision (supporting); writing – review and editing (equal). **Kehinde Obamiro:** conceptualisation (lead); funding acquisition (equal); formal analysis (supporting); methodology (supporting); supervision (lead); writing – original draft preparation (equal); writing – review and editing (equal).

## Disclosure

The authors declare the research has not been previously published in part or full elsewhere.

## Ethics Statment

Ethical approval was received from the University of Tasmania Human Research Ethics Committee (approval number: H0027256). Participants provided informed written consent.

## Conflicts of Interest

The authors declare a conflict, NG knew some participants. This was managed by another researcher (JH) conducting the interviews and de‐identified the data for these participants.

## Supporting information


Supporting File

## Data Availability

The data that support the findings of this study are available on request from the corresponding author. The data are not publicly available due to privacy or ethical restrictions.
