# Supplementary material for: Awareness and Information Seeking About Bowel Cancer and Screening in a Remote Australian Community: A Qualitative Study
Source: Health Expect. 2026 Jul 15;29(4):e70769. doi: 10.1111/hex.70769 (PMC13371089; doi:10.1111/hex.70769)
Supplement: Supplementary file 1 — Supporting File [file HEX-29-e70769-s001.docx]

**Awareness and information seeking about bowel cancer and screening in a remote Australian community: A qualitative study.**

**Supplementary Materials.**

**APPENDIX 1.** Interview topic guide for bowel cancer awareness and screening developed using the Theoretical Domains Framework version 2 and Behavioural Change Wheel.

| **Domains** | | **Questions** | **Probing questions** |
| --- | --- | --- | --- |
| **Introductory question** |  | Q. Can you tell me what you know and understand about bowel cancer? |  |
| **Screening questions** | | | |
| **CAPABILITY**  **Psychological** | ***Knowledge***  ***Memory, attention & decision processes*** | Q. Can you tell me about your experience or anything you know about bowel cancer screening? |  |
| **MOTIVATION**  **Automatic** | ***Emotion*** | Q. Can you share how you felt when you received the bowel cancer screening kit or letter? |  |
| **Screening and awareness (risk factors and symptoms) questions** | | | |
| **MOTIVATION**  **Reflective** | ***Intentions***  ***Reinforcement*** | Q. What could or has motivated you to participate in bowel cancer screening? | Motivators could be your personal beliefs, attitudes, or values.  Q. Do you see value in screening or seeking information about bowel cancer? Why or why not? |
| **CAPABILITY**  **Physical** | ***Skills*** | *Preamble: The bowel cancer screening process includes reading the instructions, using the kit, filling out the form and returning the kit in the mail. (Show participant an example screening kit).*  Q. Can you tell me about how easy or difficult it was/think it would be to undertake screening? Or to find information about bowel cancer? | Q. Where would you expect to find the information?  *(Tell participant to ensure that they are trusted sources for example government health websites or the National Bowel Cancer Screening Program website)*. |
| **OPPORTUNITY**  **Physical** | ***Environmental context & resources*** | Q. Can you think of anything in your environment that you feel would help or prevent you from taking part in bowel cancer screening? | ‘Environment’ could mean the resources in your community, your home, or your workplace. |
| ***OPPORTUNITY***  ***Social*** | ***Social influences*** | Q. Can you describe how other people may have influenced your decision to screen/or not screen? Or to seek information about bowel cancer? | ‘Other people’ might include a partner, friend, relative, health provider or community member. |
| **Awareness (risk factors and symptoms) questions** | | | |
| **CAPABILITY**  **Psychological** | ***Knowledge***  ***Behavioural regulation***    ***Memory, attention & decision processes*** | *Preamble: A symptom is an indicator that a condition or disease may be present.*  Q. Can you tell me anything you know about some of the symptoms for bowel cancer that you are aware of?  *(Tell participant other symptoms they did not mention e.g., blood in stools, diarrhoea, constipation, pain in abdomen, tiredness, unintentional weight loss)*  *Preamble: There are many things that can increase a person's chance of developing bowel cancer. They are often called 'risk factors'.*  Q. What things do you know of that increase a person’s risk of bowel cancer?  *(Tell participant other risk factors they did not mention e.g., aged over 50-years, smoker, high alcohol consumption, overweight, family history of bowel cancer, diet high in processed and red meats, low physical activity).*  Q. Can you tell me about your experience with seeking information about bowel cancer risk factors or symptoms? | Q. Where did you find out about this information? |
|  |  |  | Q. What would help ensure you seek out information about bowel cancer? |
| **MOTIVATION**  **Reflective** | ***Intention***  ***Beliefs about capabilities***  ***Optimism***  ***Beliefs about consequences*** | Q. Do you feel that you could find enough information about bowel cancer?  Q. What could or has motivated you to seek information about bowel cancer? | Q. Do you think it would be helpful to you or not to seek information about bowel cancer? |
| **Additional questions** |  | Q. Is there anything else that you think might be important for the study that we have not covered today? |  |

**APPENDIX 2.** Remote Tasmanian bowel cancer information-seeking and awareness participant questionnaire questions.

Please do not include any identifiable information (name or address) on this form.

1. What is your age?

• 50 – 54yrs

• 55 – 59yrs

• 60 – 64yrs

• 65 – 69yrs

• 70 – 74yrs

• 75yrs+

2. What is your sex?

• Female

• Male

• Non-binary sex

3. In which country were you born? _____________________

4. What language do you generally speak at home? _____________________

5. How many times have you participated in a bowel cancer screening?

• 0

• 1

• 2

• 3+

6. Have you ever undergone a colonoscopy?

• Yes

• No

• Don’t know

7. Have you ever been diagnosed with bowel cancer or bowel polyps?

• Yes

• No

• Don’t know

8. Has a family member ever been diagnosed with bowel cancer or bowel polyps?

• Yes

• No

• Don’t know

9. Do you identify as Aboriginal and/or Torres Strait Islander?

• Yes, Aboriginal

• Yes, Torres Strait Islander

• No

10. What is the highest level of education you have attained?

• Primary school or less

• High school (completed to year 10 or less)

• High school (completed to year 12)

• TAFE/Trade/vocational training

• University

11. Are you employed full-time, part-time, not employed, or retired?

• Full time

• Part time

• Not employed

• Retired

12. Including yourself, how many people live within your household?

• one

• two

• three

• four or more

Thank you for providing the above information.

**APPENDIX 3:** Codebook adapted from the Theoretical Domains Framework for Behavioural Change version 2 domains outlined in Atkins et al.^1^

| **Domain** | **Definition** | **Constructs** |
| --- | --- | --- |
| Knowledge | An awareness of the existence  of something. | Knowledge (including knowledge  of condition/scientific rationale)  Procedural knowledge  Knowledge of task environment |
| Skills | An ability or proficiency  acquired through practice. | Skills  Skills development  Competence  Ability  Interpersonal skills  Practice  Skill assessment |
| Beliefs about capabilities | Acceptance of the truth, reality  or validity about an ability, talent  or facility that a person can put to constructive use. | Self-confidence  Perceived competence  Self-efficacy  Perceived behavioural control  Beliefs  Self-esteem  Empowerment  Professional confidence |
| Optimism | The confidence that things will  happen for the best or that  desired goals will be attained. | Optimism  Pessimism  Unrealistic optimism  Identity |
| Beliefs about consequences | Acceptance of the truth, reality,  or validity about outcomes of a  behaviour in a given situation. | Beliefs  Outcome expectancies  Characteristics of outcome  expectancies  Anticipated regret  Consequents |
| Reinforcement | Increasing the probability of a  response by arranging a  dependent relationship, or  contingency, between the  response and a given stimulus. | Rewards (proximal/distal, valued/not  valued, probable/improbable)  Incentives  Punishment  Consequents  Reinforcement  Contingencies  Sanctions |
| Intentions | A conscious decision to perform  a behaviour or a resolve to act in  a certain way. | Stability of intentions  Stages of change model  Transtheoretical model and  stages of change |
| Memory, attention and  decision processes | The ability to retain information,  focus selectively on aspects of  the environment and choose  between two or more  alternatives. | Memory  Attention  Attention control  Decision making  Cognitive overload/tiredness |
| Environmental context and  resources | Any circumstance of a person’s  situation or environment that  discourages or encourages the  development of skills and  abilities, independence, social  competence and adaptive  behaviour. | Environmental stressors  Resources/material resources  Organisational culture/climate  Salient events/critical incidents  Person × environment interaction  Barriers and facilitators |
| Social influences | Those interpersonal processes  that can cause individuals to  change their thoughts, feelings,  or behaviours. | Social pressure  Social norms  Group conformity  Social comparisons  Group norms  Social support  Power  Intergroup conflict  Alienation  Group identity  Modelling |
| Emotion | A complex reaction pattern,  involving experiential,  behavioural, and physiological  elements, by which the  individual attempts to deal with  a personally significant matter  or event. | Fear  Anxiety  Affect  Stress  Depression  Positive/negative affect  Burn-out |
| Behavioural regulation | Anything aimed at managing or  changing objectively observed or  measured actions. | Self-monitoring  Breaking habit  Action planning |

**APPENDIX 4.** Themes and components of the bowel cancer awareness findings from a remote Tasmanian community.

| **Themes** | | **Enablers** | | | **Barriers** | | | | |  |
| --- | --- | --- | --- | --- | --- | --- | --- | --- | --- | --- |
|  | **Sub-themes** | | **Components** | | | **Sub-themes** | | | **Components** | |
| **Social influences enabled passive exposure to bowel cancer information and raised awareness of bowel cancer and screening.**  BCW component: Opportunity, social.  I-SAM social influencers: social support, health provider endorsement and mass media. | **Conversations with others.** | | Regularly discuss health topics with friends. | | | **Health provider competing interests in consults** | | | General practitioners are important in raising awareness but may lack time to do so in consults. | |
|  | **Know someone with bowel cancer and their experience.** | | Awareness from their family experience of bowel cancer. Some became aware or had made conclusions to some symptoms being for bowel cancer based on experiences from their family or friends. Some may not have known they were symptoms but assumed. | | |  | | |  | |
|  |  |  | Those with a family history of bowel cancer may have a higher knowledge about bowel cancer but still had gaps in their knowledge. | | |  |  |  |  | |
|  |  | | Understanding family history is a risk factor. | | |  |  |  |  | |
|  |  | | Awareness of screening from already having colonoscopies due to their family history of bowel cancer. | | |  |  |  |  | |
|  | **Existing awareness campaigns** | | Some mentioned the ‘Poo, Poke and Post’ slogan for a Tasmanian Government bowel cancer screening promotion campaign. | | | **Lack of public awareness prompts.** | | | There needs to be public awareness to make people think about bowel cancer and screening. | |
|  | **Awareness strategies are needed for younger people to be made aware of symptoms and risk factors.** | | Raising awareness earlier about general bowel cancer e.g., in younger generations so they are not complacent and can build good habits. | | |  | | | Some believe there is limited information out there for those that are not actively seeking the information. | |
|  | **Information is needed for people aged in their 40's to prepare for screening.** | | Some recommend from 30's or 40’s to have an impact on changing health habits. | | |  | | | Avoid graphs and numbers, put it in words “*Get a screen because you can treat it*.” Because you are targeting people who do not want to hear it. | |
|  |  |  | Preparing people of how and when to screen e.g., this could reduce the shock of getting the test kit in the mail at 50-years. | | |  | | |  | |
| **Environmental cues and screening materials prompted awareness, but understanding of bowel cancer and screening was variable and incomplete.**  BCW component: Opportunity, physical environment.  I-SAM physical environmental influences: prompts and invitation reminders. | **In the right environment.**  Being in the right environment can make one think more about health e.g., hospital setting. | | Many will stumble upon the information about bowel cancer rather than actively seeking the information. | | | **Lack of prompts or attentional triggers.** | | | Having the information in someone’s face a lot can make them think about it more. Otherwise, it can slip their mind. | |
|  |  |  | A job in health. | | |  | | | There needs to be prompts in people’s faces to remind them. | |
|  |  | | To pay attention to information when presented, as it can increase awareness. | | |  | | | Some do not screen or open the kit when they receive it. | |
|  | **The screening kit can be an awareness prompt.** | | The screening kit can trigger thinking about bowel cancer. | | |  | | | Bowel cancer is a taboo subject, so some do not think about it often. | |
|  |  |  | Some were able to understand symptoms from the screening process and kit instructions. | | |  | | |  | |
|  | **Information at the end of the national screening program eligibility period.** | | After people are no longer eligible to screen, there needs to be awareness about how to screen if they want to continue to do so. | | | **Lack of contact from the national screening program beyond mailing test kits.** | | | One decided years after un-registering from the national program and learning more about screening, that they want to screen but did not have prompts from program on how to re-register. | |
| **Awareness influenced screening decisions, while screening experiences also built awareness across the screening pathway.**  BCW component: Capability, psychological.  I-SAM process of decision making. | **Awareness of screening and bowel cancer can encourage/ motivate some to want to screen.** | | Awareness of the outcomes of not screening can influence one to screen. | | | **Lack of awareness can lead to ill-informed or inappropriate screening decisions.** | | | Lack of awareness of why to screen when first receiving the kit has led to people removing themselves from the national test kit register. | |
|  |  |  | Learning more about screening, can change people’s decisions to screen overtime. | | |  | |  | Low awareness of why you need to do screening when you do not have symptoms. | |
|  |  |  | Awareness of test kits available to screen for bowel cancer can encourage screening. | | |  | |  | Low awareness of risk factors may mean people feel screening is unnecessarily for them at that time e.g., age. | |
|  | **Good awareness of risk factors and symptoms can enable healthy behaviour change.** | | To be able to change towards healthier behaviours based on knowing risk factors. | | |  | | | A misconception widely that bowel cancer screening is ‘yucky’ from limited awareness of how to do the kit. Some had an inaccurate understanding of the screening process. | |
|  |  |  |  |  |  |  | | |  |  |
|  |  |  | Can enable people to take notice of symptoms if they arise. | | | **Low awareness of symptoms can be a risk factor.** | | | Lack of awareness can be a risk factor for bowel cancer as you may not know what symptoms to look for. | |
|  | **Completing screening can increase awareness of the process.** | | Awareness about the screening process can influence one's decision on the type of screening they undergo. E.g., understanding that after completing a colonoscopy, one does not need to do the next screening kit in the mail. | | | **A tendency to comply with health recommendations to screen without strong awareness of why.** | | | Some can have a limited awareness of bowel cancer and screening and still do the screening. | |
|  |  | |  | | |  | | | Lack of awareness of screening/diagnostic process may mean people screen unnecessarily e.g., complete next screening kit in mail after colonoscopy. | |
|  |  | |  | | | **Low or lack of awareness of screening options before or after national kit eligibility age.** | | | Low awareness of screening options for those not (or no longer) eligible for the national screening program. | |
|  |  | |  | | |  | | | Limited awareness of other options for where to get the screening kit e.g., not knowing one can source a kit at their general practitioner or pharmacy. | |

**APPENDIX 5.** Themes and components of the bowel cancer information seeking behaviour findings from a remote Tasmanian community.

|  | **Themes** | **Enablers** |  | **Barriers** | |
| --- | --- | --- | --- | --- | --- |
| **BCW domain** | **BCW and TDF domains** | **Sub-themes** | **Components** | **Sub-themes** | **Components** |
| **Capability** | **Capability; Physical skills.**  (Skills). | **Self-efficacy to look for information.** | Some find it easy to look for information. | **Low self-efficacy or skills to look for information.** | Some have a lack of confidence to search on the internet. |
|  |  |  | Some believe they know how to find information. |  | It could be harder for those above 70-years to seek information. |
|  |  |  |  |  | Having poor literacy skills could be a barrier to look for information. |
|  | **Capability; Psychological.** (Knowledge; cognitive and interpersonal skills; memory, attention, and decision processes; behavioural regulation). | **Lack of awareness of their own information seeking behaviours.** | Some who said they do not look for information have also mentioned in the interview times when they have looked for information. They did not think they had done it. | **Disorders/impairments.** | Reading disorders/difficulties could be a barrier to look for information from a limited ability to read. |
|  |  | **Having personal relevance may result in individuals paying more attention to information.** | Individuals may notice or pay more attention to the information available around them when they have a family history of bowel cancer or know someone close to them with bowel cancer. | **Limited awareness could result in low attention to information.** | There needs to be some awareness or something to capture peoples’ attention first, otherwise they may not take notice of it. |
|  |  |  | The decision processes of why they looked for information was because they did not want bowel cancer. |  |  |
| **Opportunity** | **Opportunity; Social.** (Social influences). | **Exposure to people with a lived experience of bowel cancer.** | Knowing someone close to them with bowel cancer e.g., family member or friend gives them a direct experience of the disease. |  |  |
|  |  |  | A job in health can expose those to others going through bowel cancer and can motivate them to look for information. |  |  |
|  |  |  | Family members or friends in a health profession or with a lived experience of bowel cancer or screening can be a resource to find the information. |  |  |
|  |  | **Social pressures.** | Family pressured them to look for the information. |  |  |
|  |  |  | General practitioner pressured them to look for information. |  |  |
|  |  | **Discussions with a health professional is a way to seek information.** | The general practitioner recommend they investigate it more and the individual has a high value for their general practitioner (without the feeling of pressure to seek information). |  |  |
|  |  |  | Having a reliable and approachable general practitioner or health provider to ask questions (information seeking via asking them questions). |  |  |
|  |  |  | Some participants think the general practitioner already encourage them in consults whereas others thought they should encourage it more. One participant who said this, also mentioned they do not generally go to the general practitioner. |  |  |
|  |  |  | Call Cancer Council to ask questions. The participant had done this for another cancer. |  |  |
|  |  | **Discussions with others (non-health professionals) is a way to seek information.** | Conversations with others. |  |  |
|  |  |  | Regularly discuss health topics with friends. |  |  |
|  |  |  | To help others. To explain to a family member who refuse to screen that it is easy and not as bad as they think. |  |  |
|  |  |  | Through discussions with their family about their risks, they decided to look for information. |  |  |
|  | **Opportunity; Physical environment.** (Environmental context and resources). | **Find information unintentionally.** | Many participants say they may just stumble upon the information about bowel cancer rather than actively seeking the information. |  |  |
|  |  |  | It can be normal in a rural town to ask one’s general practitioner casually in the street about health questions. |  |  |
|  |  |  | Printed materials: newspaper health section, health magazines, flyers, brochures. |  |  |
|  |  |  | Health television shows or advertisements, or radio segments. |  |  |
|  |  | **Find information intentionally.** | Internet: through either reputable sites or the first suggestions from the search were both suggested as where participants would look. |  |  |
|  |  |  | Internet, non-reputable sites: Participants are not necessarily looking for the reputable sites for the information. Some just look at the first site that comes up. |  |  |
|  |  |  | Printed materials: general practitioner waiting room (pamphlets, flyers). Having information available in front of them in general practitioner waiting room, they like to read the information around them. |  |  |
|  |  |  | A health provider: Better to go to the general practitioner rather than trusting internet sources. |  |  |
|  |  |  | Some participants look for information about bowel cancer as part of their job, discussing with colleagues. They are not generally looking for themselves, it is more for discussions with others. |  |  |
|  |  |  | They feel healthy and have not thought about it previously until they are triggered by the screening kit in the mail. |  |  |
| **Motivation** | **Motivation; Automatic.** (Reinforcement; emotion). | **Interest in health.** | Enjoy reading about health. Not necessarily looking for bowel cancer specific information. | **Denial or do not want to know.** | Some would rather not know about bowel cancer. |
|  |  |  | A job in the healthcare setting can have a general interest in health. | **Potential stigma around looking for information about bowel cancer.** | If there were more positive attitudes towards bowel cancer, individual’s may be more inclined to look for information. |
|  |  | **Their age.** | A motivator for one participant to look for information. |  | Younger generations (50's) may think it is taboo to discuss bowel cancer and screening. |
|  |  | **To help others.** | A job in health, to help others. To explain to others about it being treatable and to reduce their fears. |  | Older generations can be more private about their health. |
|  | **Motivation; Reflective.** (Social/professional role and identity; beliefs about capabilities; optimism; intentions; goals; beliefs about consequences). | **Personal relevance: through having a symptom or bowel cancer.** | Most participants stated it may not be helpful to look for information about bowel cancer but also acknowledge they would look for it if they felt they needed more information on it e.g., if they thought they had symptoms or cancer itself. | **Lack of personal relevance.** | Rely on screening e.g., believe it is not necessary to look for information or they do not look as they regularly screen already and have no symptoms. |
|  |  |  | If they think they have a symptom of bowel cancer, it could motivate them. |  | No symptoms present, so do not need to look. |
|  |  |  | Had a previous positive test result from the screening kit. |  | Have not had a positive test result previously. |
|  |  |  | If they had bowel cancer. |  | Feel healthy, so do not feel the need to look for the information. |
|  |  |  | If their family had bowel cancer. One participant believes it is more important to look for information on screening than bowel cancer itself as they believe they are healthy and may not change their ways. | **Want to focus on living a healthy life, not just on one type of cancer.** | More intentions to just live a healthy life for overall health and reduce the risk of all cancers rather than worrying about looking for information about one specific cancer. |
|  |  |  | **Have a family history:** Want to avoid getting bowel cancer. | **Trust that it will be managed by health providers.** | Believe the health professionals take care of that and will tell them what they need to know. They will just do what the professionals tell them to do. |
|  |  | **To make the best choices for their health.** | Believe it is useful to look for information as they see value in information and knowing more about bowel cancer. | **Relying on family experiences.** | They are happy to just go off family experience and not seek the information. |
|  |  |  | To feel they have the control over their own health and the potential health outcomes from their choices. | **Lack of interest to look for information.** | You cannot force individuals to look for information, they need to want to do it. |
|  |  |  |  |  | Can have self-efficacy to look for information but lack interest. |
|  |  |  |  |  | Need to see the value in looking for the information on bowel cancer. |
|  |  |  |  |  | Some said it would be useful to look for information but do not intend to look themselves. |

1. Atkins L, Francis J, Islam R, O'Connor D, Patey A, Ivers N, et al. A guide to using the Theoretical Domains Framework of behaviour change to investigate implementation problems. *Implementation Science*. 2017;12(1):77.10.1186/s13012-017-0605-9
